# Observations on Indigenous Medical Plants

**Published:** 1856-10

**Authors:** Ariel Hunton

**Affiliations:** Hyde Park, Vt.


*Observations on Indigenous Medical Plants.* By Ariel Hunton, M. D., Hyde Park, Vt.

Since my last paper on the medical virtues of our indigenous vegetables, I have selected a few more, which I will describe:

*Erechthites hieracifolia,* the Senecio hieracifolius of Linnæus; *first growth fireweed,* belonging to the class Syngenesia; order, Superflua. An annual grooved, juicy, herbaceous stem, abundant on recently burned lands. It is aromatic, and has a slightly fetid odor. The most use I have made of the article, is to direct patients troubled with hemorrhoids, to simmer the stalks and leaves in lard, and apply the ointment in this disease. It usually gives relief.

An essential oil is extracted, and used for the same purpose. It possesses alterative properties, and is used as a domestic remedy in the summer complaints of children.

*Epilobium angustifolium.*—*Second growth fireweed,* wicapy, rosebag, &c. This article was omitted in my description of vesicatories, for the very good reason that I could not class it until it was in blossom; the class is Octandria; order, Monogynia; it grows to the height of four to six feet; the flowers are showy, of a pink color; in a long spike; will be in blossom from the first of July to October; the capsule is two to three inches long, about the bigness of a darning-needle; the seeds armed with an egret. The recent root, bruised, is used as a counter-irritant, and will vesicate; a decoction of the root and leaves is used in diarrhœa, dysentery, and leucorrhœa, as a tonic and astringent.

*Menyanthes trifoliata,* buck-bean, marsh-trefoil; class, Pentandria; order, Monogynia. There are in this vicinity several bogs where this article grows in abundance; one gill of an infusion, of half an ounce of the root to one pint of water, will usually operate as a cathartic and emetic. In small doses it is in use as a tonic and alterative in scrofulous, and most cachectic affections.

*Tiarella cordifolia,* miterwort, coalwort; class, Decandria; order, Digynia. An infusion, in cold or warm water, makes a palatable febrifuge. I have used lhe article more than forty years.

*Saponaria officinalis,* soapwort, bouncing-bet; class, Decandria; order, Digynia. Soapwort is an alterative, used in strumous and skin diseases; antisyphilitic in gonorrhœa and gleet; will excite the torpid liver, and stimulate the bile ducts; the extract is in use; dose, from grs 10 to 20.

*Polytrichum juniperum,* hair-cap moss, bear's bed; class, Cryptogamia; order, Musci. This article, not having a place in the *U. S. Dispensatory,* and being a useful ingredient, is more used in domestic practice than by the faculty; I give it a passing notice.

Our botanists, in their descriptions, locate it in dry woods; it is abundant on all our poor knolls, in pastures, and waste uncultivated lands.

It is quite a sure diuretic, and has been used in decoction, as a domestic remedy in what the people denominate the *gravel,* by which they mean disury, or any disease of the urinary organs.

When I am treating a disease of the above description, the friends of the patient will recommend a decoction of bear's-bed, and extol its powers as a diuretic. Being always willing to witness the effect of our indigenous vegetables, I have frequently seen marked effects from its exhibition, relieving the pain and anguish, and causing an abundant flow of urine.

